# Toll-like receptor 4 Asp299Gly and Thr399Ile polymorphisms: New data and a meta-analysis

**DOI:** 10.1186/s12876-014-0206-x

**Published:** 2014-12-10

**Authors:** Nezha Senhaji, Brehima Diakité, Nadia Serbati, Younes Zaid, Wafaa Badre, Sellama Nadifi

**Affiliations:** Laboratory of Genetic and Molecular Pathology (LGPM), Medical School, Hassan II University, Casablanca, Morocco; Gastroenterology Department, CHU Ibn Rochd, Casablanca, Morocco; Laboratory of Thrombosis and Haemostasis Research Centre, Montreal Heart Institute, 5000 Belanger Street, Montreal, QC H1T 1C8 Canada

**Keywords:** Toll-like receptor 4, Inflammatory bowel disease, Moroccan patients

## Abstract

**Background:**

The pathogenesis of inflammatory bowel disease (IBD) involves interactions between the host genetic susceptibility, intestinal microflora and mucosal immune responses through the pattern recognition receptor. Polymorphisms in toll-like receptor 4 (*TLR4*) induce an aberrant immune response to indigenous intestinal flora, which might favor IBD development. In this study, we aimed to determine whether *TLR4* gene was associated with Crohn’s disease (CD) and ulcerative colitis (UC) among Moroccan patients, and evaluated its correlation with clinical manifestation of the disease.

**Methods:**

The study population comprised 117 patients with IBD and 112 healthy unrelated blood donors. *TLR4* polymorphisms: Asp299Gly and Thr399Ile were genotyped by polymerase chain reaction-restriction fragment length polymorphism. PCR products were cleaved with Nco I for the Asp299Gly polymorphism and Hinf I for the Thr399Ile polymorphism. Meta-analysis was performed to test the association of 299Gly and 399Ileu carriage with CD, UC and the overall IBD risk.

**Results:**

Our study revealed that the frequency of Asp299Gly and Thr399Ile did not differ significantly between patients and controls in the Moroccan population. However, meta-analysis demonstrated significantly higher frequencies of both Asp299Gly and Thr399Ile SNPs in IBD and CD and for 399Ileu carriage in UC patients.

**Conclusion:**

The meta-analysis provides evidence that *TLR4* polymorphisms confer a significant increased risk for the overall IBD development.

## Background

Inflammatory bowel disease (IBD) is an idiopathic and chronic multifactorial disease of the gastrointestinal tract. Although the precise etiology of IBD is unclear, several factors that play a crucial role in disease pathogenesis such as commensal bacterial flora and genes related to the host immune response have been identified [1,2].

Toll-like receptors are pattern recognition receptors through which host recognizes microbial conserved molecular motifs that are broadly shared by pathogens, therefore they are very important for the regulation of mucosal innate immune responses to intestinal microbes. Perturbations in individual TLR biological signaling can prime to a number of different outcomes and elucidate a system of regulation within the intestine in which each TLR plays a largely non-redundant role in mucosal immunity.

*TLR4* gene, the first mammalian TLR identified is located on the long arm of human chromosome 9q32-33 [3]. It encodes the transmembrane receptor that initiates the innate immune response to common gram-negative bacteria [4,5].

TLR4 is the major transducer of lipopolysaccharide (LPS) and binds specifically lipid A moiety. Signal transduction through TLR4 in combination with CD14, and MD-2 leads to activation of the nuclear factor-κB (NF-κB) system through the MyD88-dependent and MyD88-independent pathways and subsequent expression of inflammatory genes encoding cytokines and cell conjugation molecules as part of host defense mechanisms [6-9].

Under healthy conditions TLR4 is only minimally expressed in lamina propria mononuclear cells (LPMNCs) and intestinal epithelial cells which are partly tolerant to LPS, thus preventing an exaggerated immune response mediated by the large number of bacteria in the intestinal lumen and maintaining a basal state of activation [10,11].

However, TLR4 expression is upregulated in human IBD colitis, maximizing responsiveness to the environment and reflecting an aberrant state of activation [12-15]. Higher levels of TLR4 mRNA and protein were found in the inflamed colonic mucosa in pediatric IBD patients [16]. Two common co-segregating polymorphisms affecting the extracellular domain of the *TLR4* (Asp299Gly and Thr399Ile) have been described in humans. Individuals heterozygous for these mutations have a blunted response to inhaled LPS [17]. These polymorphisms are thought to be associated with increased susceptibility to IBD. However, population studies reveal discordant results in geographical distribution.

Thereby, we sought to investigate whether Asp299Gly and Thr399Ile single nucleotide polymorphisms of the gene encoding the *TLR4* determine susceptibility to IBD in Moroccan patients and assessed their influence on phenotype expression.

## Methods

### Study population

Enrolled in this study were 117 IBD Moroccan patients (83 CD; 34 UC) and 112 healthy unrelated blood donors. The diagnosis of CD or UC was established according to conventional clinical, endoscopic, radiological and histological criteria as previously reported [18,19]. CD was classified according to the Montreal classification [20].

The case report form included questions on disease phenotype and location, age at diagnosis, toxic behavior and other clinical features. The ethics committee of the Faculty of Medicine and Pharmacy of Casablanca approved the study and a written informed consent was obtained from all human subjects.

### Molecular analysis of *TLR4* polymorphisms

Genomic DNA was extracted from peripheral blood leukocytes using the salting out procedure. Two single nucleotide variations, corresponding to two amino acid polymorphisms for *TLR4*, were analyzed: the Asp299Gly (896A/G), rs4986790 and the Thr399Ile (1196C/T), rs4986791.

Typing of the polymorphisms was performed using polymerase chain reaction (PCR) restriction fragment length polymorphism analysis (RFLP).

Upstream and downstream primers used for the PCR amplification were:

- F: (5′- AGCATACTTAGACTACTACCTCCATG-3′),
- R: (5′- GAGAGATTTGAGTTTCAATGTGGG-3′) for *TLR4Asp299Gly*
- And F: (5′-GGTTGCTGTTCTCAAAGTGATTTTGGGAGAA-3′),
- R:(5′-GGAAATCCAGATGTTCTAGTTGTTCTAAGCC-3′) for *TLR4Thr399Ile.*

Reactions were performed in a 25 μl volume containing 200 μM of each dNTP (0.5 μl of dNTP mix, 10 mM each), 0.2 μM of each of the forward and reverse primers (0.5 μl of each 10 μM primers), 2 mM MgCl2 (1 μl of MgCl2, 50 mM) and 1 U of Taq DNA polymerase (1 μl of 1U/μl enzyme), 1× PCR buffer (2.5 μl of 10× PCR buffer).

PCR conditions comprised 5 min at 95°C then 35 cycles of denaturing were performed at 95°C for 30 s, annealing at 55°C (Asp299Gly) and at 53°C (Thr399Ile) for 1 min, 72°C for 30 s. A final extension phase of 72°C for 10 minutes was used.

PCR products were cleaved overnight at 37°C with Nco I for Asp299Gly polymorphism and HinfI for Thr399Ile polymorphism (Biolabs). The digests were run on a 3% agarose gel and visualized under UV light using ethidium bromide.

The mutant alleles (GG)/(TT) contained an Nco I/ Hinf I restriction site for the Asp299Gly/Thr399Ile polymorphisms respectively, allowing RFLP analysis of the digested products. Digestion at the Nco I site yields fragments of 168 and 20 bp, the one at Hinf I site yields fragments of 98 and 26 bp. The wild-type allele for both polymorphisms remained uncut.

### Statistical analysis

The data were analyzed with MedCalc 11.6. Chi-square test was used to compare the allele and genotype frequencies between disease and control groups. The Fisher’s exact test was used when appropriate. The observed genotype frequencies were compared with the predicted frequencies by the Hardy–Weinberg equilibrium.

The average age was determined by the rank sum test. Associations between genotypes and risk of IBD were estimated by calculating odds ratio (OR) with confidence interval of 95% (CI). *P* values less than 0.05 were considered significant in disease risk association tests. The χ2 test or Fisher test was used to correlate the *TLR4* polymorphisms and clinical parameters. The Bonferroni correction method was applied for correction for multiple testing in sub-phenotype analysis; The phenotype genotype correlation was considered statistically significant if the *p* value was less than 0.005 for CD and 0.007 for UC. According to Power Calculator for Genetic Studies 2006 software (http://www.sph.umich.edu/csg/abecasis/CaTS/), this study had 15% of power to detect an OR of 1.5.

### *TLR4* meta-analysis

#### Inclusion and exclusion criteria

Genetic association studies were included in our meta-analysis if they met the following criteria:

(a) Studies that evaluated the association between the *TLR4* Asp299Gly, Thr399Ile polymorphisms and IBD, (b) A case–control study design, (c) The study reported sufficient data to calculate allele frequencies, odds ratios and confidence intervals of cases and controls for carriage of the *TLR4* 299Gly and 399Ile alleles.

While major exclusion criteria were: (a) case-only study and review articles (b) absence of the mutant allele in both cases and controls, (c) studies without the raw data of the *TLR4* Asp299Gly and Thr399Ile genotypes.

### Pooled studies for case–control meta-analysis

Twenty-four case–control studies were identified through the literature search.

- ***Asp299Gly polymorphism:*** According to the inclusion criteria, twenty studies were retrieved in CD meta-analysis (Table 1), four of them contained more than one cohort [21-24]. UC meta-analysis reported data from 13 of the included studies (Table 2); two of them contained more than one cohort [21,22]. Six studies met one of the exclusion criteria [25-30].
- ***Thr399Ile Polymorphism:*** twelve studies comprising 2466 cases and 2210 controls were included in CD meta-analysis (Table 3), and nine of them in UC meta-analysis with 1358 cases and 1773 controls (Table 4).
  A meta-analysis combining CD and UC patients for the two tested SNPs: Asp299Gly and Thr399Ile included 13 and 9 studies respectively (Tables 5 and 6).
  The risk of IBD associated with the *TLR4* polymorphism was estimated for each study by odds ratio (OR) and 95% confidence interval (95% CI).
  The meta-ORs were estimated using a fixed-effects model with the wild-type allele as reference group. Genetic heterogeneity was tested by Cochran’s (Q) test, I^2^ statistics was used to quantify the between-study heterogeneity effect. When a significant Q test (Q > 0.10; I^2^ > 50%) indicated heterogeneity across studies, data were recombined using a random-effects model to estimate common ORs. The meta-analyses were conducted by Review Manager 5.0 and MedCalc bvba 12.3.0 softwares.

**Table 1:** **Pooled analysis of studies exploring the role of TLR4 Asp299Gly in CD**

**Study**	**Cases (Events/Total)**	**Controls (Events/Total)**	**Odds ratio**	**95% CI**	**P value**
**Arnott et al. 2004** **[** 31 **]**	50/468	33/378	1,25	0,79 to 1,98	
**Franchimont et al. 2004** **[** 32 **]** **(1)**	73/668	14/278	2,31	1,28 to 4,17	
**Franchimont et al. 2004** **[** 32 **]** **(2)**	26/226	14/278	2,45	1,25 to 4,81	
**Torok et al. 2004** **[** 33 **]**	14/204	12/290	1,70	0,77 to 3,77	
**Braat et al. 2005** **[** 34 **]**	68/822	13/274	1,81	0,98 to 3,33	
**Brand et al. 2005** **[** 22 **]**	29/408	15/398	1,95	1,03 to 3,70	
**Lakatos et al. 2005** **[** 35 **]**	104/1054	48/400	0,80	0,56 to 1,15	
**Gazouli et al. 2005** **[** 36 **]**	19/240	6/200	2,78	1,09 to 7,10	
**Oostenbrug et al. 2005** **[** 37 **]**	53/786	27/592	1,51	0,94 to 2,43	
**Ouburg et al. 2005** **[** 38 **]**	23/224	18/340	2,04	1,08 to 3,88	
**Fries et al. 2005** **[** 39 **]** **(1)**	2/46	2/118	2,63	0,36 to 19,29	
**Fries et al. 2005** **[** 39 **]** **(2)**	10/120	2/118	5,27	1,13 to 24,60	
**Zouiten-Mekki et al. 2009** **[** 40 **]**	12/180	9/160	1,19	0,49 to 2,92	
**Hong et al. 2007** **[** 41 **]**	26/364	32/376	0,82	0,48 to 1,42	
**Baumgart et al. 2007** **[** 21 **]** **(1)**	6/288	16/404	0,51	0,20 to 1,33	
**Baumgart et al. 2007** **[** 21 **]** **(2)**	28/482	49/806	0,95	0,59 to 1,54	
**Browning et al. 2007** **[** 42 **]**	50/778	44/832	1,23	0,81 to 1,86	
**De Ridder et al. 2007** **[** 23 **]** **(1)**	11/144	20/488	1,93	0,91 to 4,14	
**De Ridder et al. 2007** **[** 23 **]** **(2)**	63/756	20/488	2,12	1,27 to 3,56	
**Riis et al. 2007** **[** 43 **]**	32/422	152/1236	0,58	0,39 to 0,87	
**Hume et al. 2008** **[** 44 **]**	87/1238	36/720	1,43	0,96 to 2,14	
**Rigoli et al. 2008** **[** 45 **]**	10/266	8/206	0,96	0,38 to 2,49	
**Manolakis et al. 2013** **[** 46 **]**	20/326	33/548	1,02	0,58 to 1,81	
**Current study 2014**	9/166	10/224	1,22	0,49 to 3,09	
**Total (fixed effects)**	825/10676	633/10152	1,26	1,13 to 1,42	0.0001
**Total (random effects)**	825/10676	633/10152	1,35	1,12 to 1,64

**Table 2:** **Pooled analysis of studies exploring the role of TLR4 Asp299Gly in UC**

**Study**	**Cases (Events/Total)**	**Controls (Events/Total)**	**Odds ratio**	**95% CI**	**P value**
**Arnott et al. 2004** **[** 31 **]**	35/492	33/378	0,801	0,49 to 1,31	
**Franchimont et al. (1) 2004** **[** 32 **]**	32/326	14/278	2,052	1,07 to 3,93	
**Torok et al. 2004** **[** 33 **]**	18/196	12/290	2,343	1,10 to 4,98	
**Braat et al. 2005** **[** 34 **]**	24/452	13/274	1,126	0,56 to 2,25	
**Gazouli et al. 2005** **[** 36 **]**	6/170	6/200	1,183	0,37 to 3,73	
**Oostenbrug et al. 2005** **[** 37 **]**	21/358	27/592	1,304	0,72 to 2,34	
**Baumgart et al. 2007** **[** 21 **]** **(1)**	8/236	16/404	0,851	0,36 to 2,02	
**Baumgart et al. 2007** **[** 21 **]** **(2)**	24/290	49/806	1,394	0,84 to 2,31	
**Browning et al. 2007** **[** 42 **]**	51/810	44/832	1,203	0,79 to 1,82	
**Riis et al. 2007** **[** 43 **]**	53/808	152/1236	0,501	0,36 to 0,69	
**De Ridder et al. 2007** **[** 23 **]** **(1)**	33/452	20/488	1,843	1,04 to 3,26	
**De Ridder et al. 2007** **[** 23 **]** **(2)**	4/62	20/488	1,614	0,53 to 4,88	
**Rigoli et al. 2008** **[** 45 **]**	3/90	8/206	0,853	0,22 to 3,29	
**Manolakis et al. 2013** **[** 46 **]**	41/374	33/548	1,921	1,19 to 3,10	
**Current study 2014**	6/68	10/224	2,071	0,72 to 5,92	
**Total (fixed effects)**	359/5184	457/7244	1,092	0,94 to 1,26	0.20
**Total (random effects)**	359/5184	457/7244	1,268	0,95 to 1,69

**Table 3:** **Pooled analysis of studies exploring the role of TLR4 Thr399Ile in CD**

**Study**	**Sample size**	**Cases (Events/Total)**	**Controls (Events/Total)**	**Odds ratio**	**95% CI**	**P value**
**Torok et al. 2004** **[** 33 **]**	CD: 102	16/204	12/290	1.972	0.91 to 4.26	
	HC: 145					
**Braat et al. 2005** **[** 34 **]**	CD: 204	30/408	19/398	1.583	0.87 to 2.86	
	HC: 199					
**Gazouli et al. 2005** **[** 36 **]**	CD: 120	1/240	2/200	0.414	0.037 to 4.60	
	HC: 100					
**Oostenbrug et al. 2005** **[** 37 **]**	CD: 393	69/1008	29/598	1.442	0.92 to 2.25	
	HC: 296					
**Zouiten-Mekki et al. 2009** **[** 40 **]**	CD: 90	13/180	8/160	1.479	0.59 to 3.66	
	HC: 80					
**Hong et al. 2007** **[** 41 **]**	CD: 182	30/364	32/376	0.966	0.57 to 1.62	
	HC: 188					
**Browning et al. 2007** **[** 42 **]**	CD: 389	47/778	46/832	1.099	0.72 to 1.67	
	HC: 416					
**De Ridder et al. 2007** **[** 23 **]**	CD: 450	72/900	22/488	1.842	1.12 to 3.00	
	HC: 244					
**Rigoli et al. 2008** **[** 45 **]**	CD: 133	8/266	6/206	1.034	0.35 to 3.02	
	HC:103					
**Azzam et al. 2012** **[** 47 **]**	CD: 46	26/92	22/100	1.397	0.72 to 2.69	
	HC: 50					
**Manolakis et al. 2013** **[** 46 **]**	CD: 163	20/326	33/548	1.020	0.57 to 1.80	
	HC: 274					
**Our study 2014**	CD: 83	7/166	3/224	3.243	0.82 to 12.73	
	HC: 112					
**Total (fixed effects)**		339/4932	234/4420	1.345	1.12 to 1.60	0.002
**Total (random effects)**		339/4932	234/4420	1.336	1.11 to 1.59

**Table 4:** **Pooled analysis of studies exploring the role of TLR4 Thr399Ile in UC**

**Study**	**Sample size**	**Cases (Events/Total)**	**Controls (Events/Total)**	**Odds**	**95% CI**	**P value**
**Torok et al. 2004** **[** 33 **]**	UC: 98	22/196	12/290	2.929	1.41 to 6.06	
	HC: 145					
**Gazouli et al. 2005** **[** 36 **]**	UC: 85	3/170	2/200	1.778	0.29 to 10.77	
	HC: 100					
**Oostenbrug et al. 2005** **[** 37 **]**	UC: 179	24/434	19/598	1.784	0.96 to 3.30	
	HC: 296					
**Zouiten-Mekki et al. 2009** **[** 40 **]**	UC: 30	2/60	2/160	2.724	0.37 to 19.78	
	HC: 80					
**Browning et al. 2007** **[** 42 **]**	UC: 405	59/810	46/832	1.342	0.90 to 1.99	
	HC: 416					
**De Ridder et al. 2007** **[** 23 **]**	UC: 257	34/514	22/488	1.500	0.86 to 2.60	
	HC: 244					
**Rigoli et al. 2008** **[** 45 **]**	UC: 45	3/90	6/206	1.149	0.28 to 4.70	
	HC:103					
**Manolakis et al. 2013** **[** 46 **]**	UC: 187	41/374	33/548	1.921	1.19 to 3.10	
	HC: 274					
**Our study 2014**	UC: 34	3/68	3/224	3.400	0.67 to 17.25	
	HC: 112					
**Total (fixed effects)**		191/2716	145/3546	1.695	1.35 to 2.11	0.0001
**Total (random effects)**		191/2716	145/3546	1.699	1.35 to 2.12

**Table 5:** **Pooled analysis of studies exploring the role of TLR4 Asp299Gly in IBD**

**Study**	**Sample size**	**Cases (Events/Total)**	**Controls (Events/Total)**	**Odds ratio**	**95% CI**	**P value**
**Arnott et al. 2004** **[** 31 **]**	IBD: 480	85/960	33/378	1,016	0,667 to 1,54	
	HC: 189					
**Franchimont et al. 2004** **[** 32 **]**	IBD: 610	131/1220	14/278	2,268	1,286 to 4,00	
	HC: 139					
**Torok et al. 2004** **[** 33 **]**	IBD: 200	32/400	12/290	2,014	1,019 to 3,98	
	HC: 145					
**Braat et al. 2005** **[** 34 **]**	IBD: 637	92/1274	13/274	1,563	0,861 to 2,83	
	HC: 137					
**Gazouli et al. 2005** **[** 36 **]**	IBD: 205	25/410	6/200	2,100	0,847 to 5,20	
	HC: 100					
**Oostenbrug et al. 2005** **[** 37 **]**	IBD: 572	74/1144	27/592	1,447	0,921 to 2,27	
	HC: 296					
**Baumgart et al. 2007** **[** 21 **]** **(1)**	IBD: 262	14/524	16/404	0,666	0,321 to 1,38	
	HC: 202					
**Baumgart et al. 2007** **[** 21 **]** **(2)**	IBD: 386	52/772	49/806	1,116	0,745 to 1,67	
	HC: 403					
**Browning et al. 2007** **[** 42 **]**	IBD: 796	101/1592	44/832	1,213	0,843 to 1,74	
	HC: 416					
**Riis et al. 2007** **[** 43 **]**	IBD: 615	85/1230	152/1236	0,529	0,401 to 0,69	
	HC: 618					
**De Ridder et al. 2007** **[** 23 **]**	IBD: 103	15/206	20/488	1,838	0,921 to 3,66	
**De Ridder et al. 2007** **[** 23 **]**	IBD: 604	96/1208	20/488	2,020	1,233 to 3,31	
	HC: 244					
**Rigoli et al. 2008** **[** 45 **]**	IBD: 178	13/356	8/206	0,938	0,382 to 2,30	
	HC: 103					
**Manolakis et al. 2013** **[** 46 **]**	IBD: 350	61/700	33/548	1,490	0,960 to 2,31	
	HC: 274					
**Our study 2014**	IBD:117	15/234	10/224	1,466	0,644 to 3,33	
	HC:112					
**Total (fixed effects)**		891/12230	457/7244	1,154	1,021 to 1,30	0.015
**Total (random effects)**		891/12230	457/7244	1,306	1,006 to 1,69

**Table 6:** **Pooled analysis of studies exploring the role of TLR4 Thr399Ile in IBD**

**Study**	**Sample size**	**Cases (Events/Total)**	**Controls (Events/Total)**	**Odds**	**95% CI**	**P value**
**Torok et al. 2004** **[** 33 **]**	IBD: 200	38/400	12/290	2.432	1.24 to 4.74	
	HC: 145					
**Gazouli et al. 2005** **[** 36 **]**	IBD: 205	4/410	2/200	0.975	0.17 to 5.37	
	HC: 100					
**Oostenbrug et al. 2005** **[** 37 **]**	IBD: 721	93/1442	29/598	1.353	0.88 to 2.07	
	HC: 299					
**Zouiten-Mekki et al. 2009** **[** 40 **]**	IBD: 120	15/240	8/160	1.267	0.52 to 3.06	
	HC: 80					
**Browning et al. 2007** **[** 42 **]**	IBD: 794	106/1588	46/832	1.222	0.85 to 1.74	
	HC: 416					
**De Ridder et al. 2007** **[** 23 **]**	IBD: 707	106/1414	22/488	1.717	1.07 to 2.75	
	HC: 244					
**Rigoli et al. 2008** **[** 45 **]**	IBD: 178	12/356	6/206	1.163	0.43 to 3.14	
	HC: 103					
**Manolakis et al. 2013** **[** 46 **]**	IBD: 350	61/700	33/548	1.490	0.96 to 2.31	
	HC: 274					
**Our study 2014**	IBD: 117	10/234	3/224	3.289	0.89 to 12.11	
	HC: 112					
**Total (fixed effects)**		445/6784	161/3546	1.479	1.22 to 1.82	0.0001
**Total (random effects)**		445/6784	161/3546	1.465	1.21 to 1.80	

## Results

Hundred and seventeen patients with IBD (83 CD; 34 UC) and 112 control subjects from the general population were genotyped for the presence of *TLR4*Asp299Gly and Thr399Ile polymorphisms.

The average age of CD, UC patients and controls was 27.6 ± 2.3, 40 ± 5.0 and 31.3 ± 2.1 years respectively. The distributions of genotype and allele frequencies of both *TLR4*Asp299Gly and Thr399Ile polymorphisms in CD patients (X2 = 0.03, P = 0.86; X2 = 0.02, P = 0.90) and healthy controls (X2 = 2.86, P = 0.24; X2 = 0.01, P = 0.94) were in Hardy-Weinberg equilibrium. In patients with UC, genotype and allele frequencies distributions for Asp299Gly polymorphism (X2 = 0.03, P = 0.86) were in Hardy-Weinberg equilibrium but not for Th399ILeu polymorphism (X2 = 19.05, P <0.001).

In order to study associations of *TLR4* variants in IBD overall and in CD and UC in particular, the distribution of *TLR4* polymorphic alleles was assessed. Genotype and allele frequencies are given in Table 7 and genotypic and allelic odds ratios and test P-values are presented in Table 8. None CD nor UC colitis patients were homozygous for G allele. Mutant allele frequency was 5.4% in CD, 8.8% in UC and 4.5% in HC. No significant difference was noticed in allele distributions of the Asp299Gly polymorphism between the control and patient groups. Likewise, no significant association of IBD with the Thr399Ile polymorphism was found in either cohort (allele frequencies: HC 1.3%, CD 4.2%, UC 4.4%). TT genotype was not observed in both CD patients and HC and only one individual carried the 399Ile variant at both alleles in UC. Co-segregation of *TLR4* polymorphic alleles was observed in only 33% of controls (3 out of 9), 33% in UC (2 out of 6) and 60% in CD (6 out of 10).

**Table 7:** **Allele and genotype frequencies of the studied polymorphisms in the group of patients with Crohn’s disease, ulcerative colitis and controls**

**Group**	**TLR4 Asp299Gly**						**TLR4 Thr399Ile**			
	**A**	**G**	**AA**	**AG**	**GG**	**C**	**T**	**CC**	**CT**	**TT**
**CD (%) N = 83**	157 (94.6)	9 (5.4)	74 (89.2)	9 (10.8)	-	159 (95.8)	7 (4.2)	76 (91.6)	7 (8.4)	-
**UC (%) N = 34**	62 (91.2)	6 (8.8)	28 (82.4)	6 (17.6)	-	65 (95.6)	3 (4.4)	32 (94.1)	1 (2.9)	1 (2.9)
**Controls (%) N = 112**	214 (95.5)	10 (4.5)	103 (92.0)	8 (7.1)	1 (0.9)	221 (98.7)	3 (1.3)	109 (97.3)	3 (2.7)	-

**Table 8:** **Odds ratios and P values for association of TLR4 variants with IBD status**

**SNP**	**Trait**	**Genotype/Allele**	**OR**	**CI**	**P Value**
**Asp299Gly**	**CD**	**AG**	1.57	(0.58-4.25)	0.38
		**G**	1.23	(0.49-3.09)	0.66
	**UC**	**AG**	2.76	(0.88-8.61)	0.08
		**G**	2.07	(0.72-5.92)	0.17
**Thr399Ile**	**CD**	**CT**	3.35	(0.84-13.35)	0.09
		**T**	3.24	(0.83-12.74)	0.09
	**UC**	**CT**	1.14	(0.11-11.29)	0.91
		**TT**	10.11	(0.40-254.1)	0.16
		**T**	3.4	(0.67-17.25)	0.14

**Figure 1:**
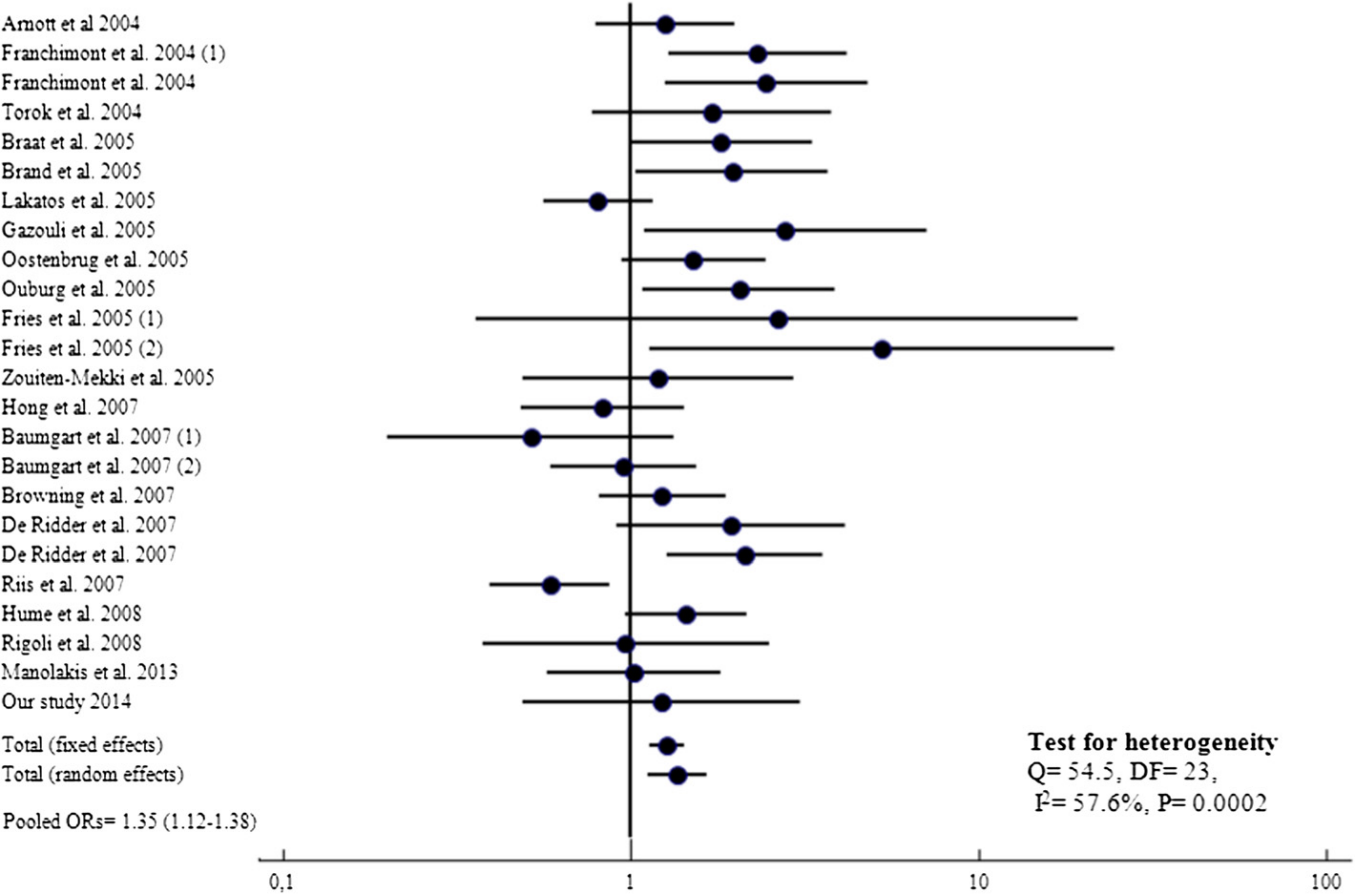
**Forest plots for the association of TLR4 A299G and risk of CD.**

**Figure 2:**
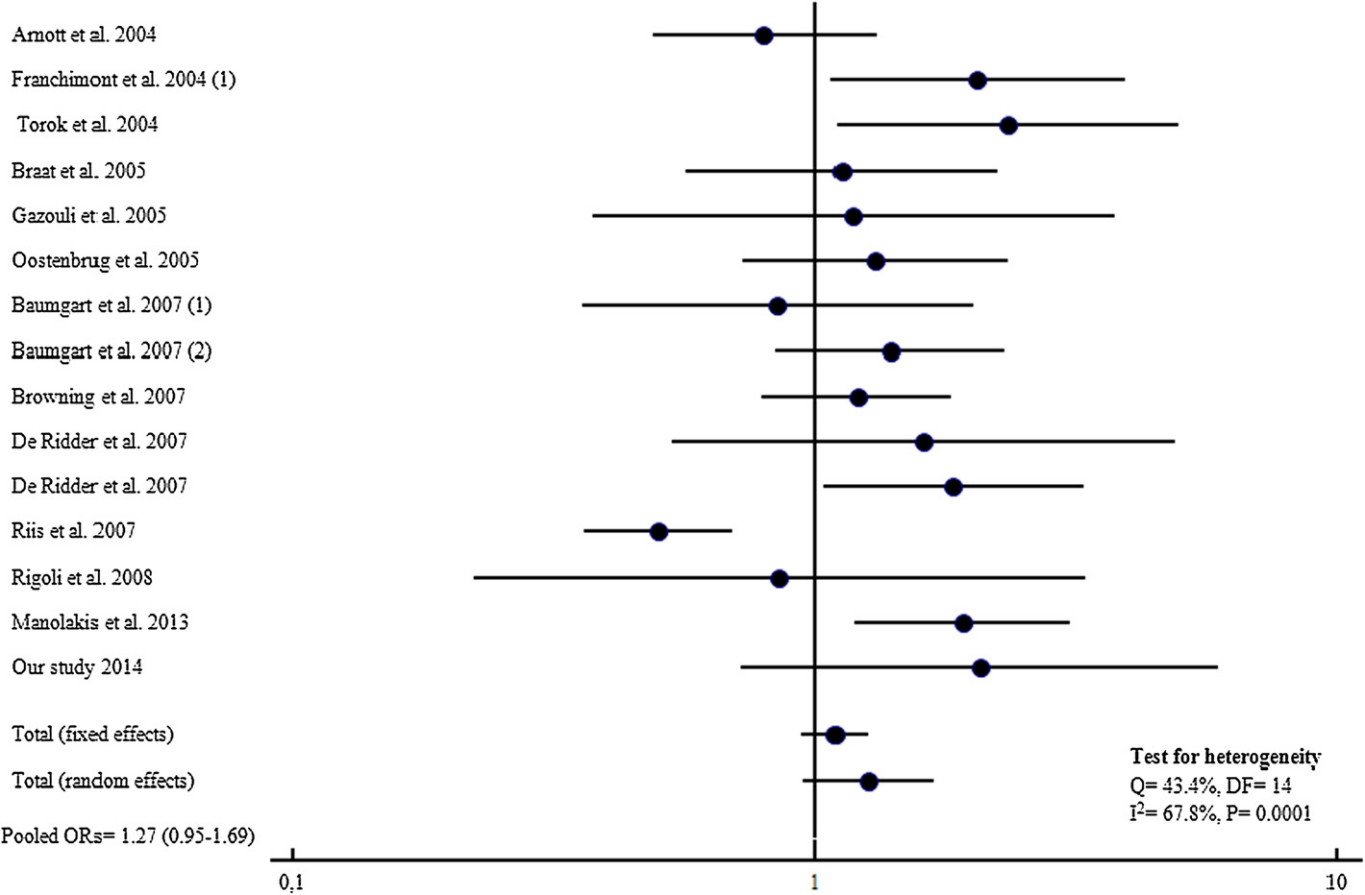
**Forest plots for the association of TLR4 A299G and risk of UC.**

Meta-analysis of our dataset with the published studies showed a significant association between *TLR4* Asp299Gly variant allele and CD risk in a total of 5338 cases and 5076 controls (Pooled ORs = 1.35, 95% CI: 1.12-1.38; P = 0.0001) (Figure 1). In the other hand, no association with UC was found when evaluating disease risk in 2592 patients and 3622 controls (Table 2), OR = 1.27, 95% CI = 0.95-1.69; P = 0.20 (Figure 2). Heterogeneity in odds ratios between studies was evidenced for CD (Q = 54.5, 23 df, *P* = 0.0002, I^2^ = 57.6%) and UC (Q = 43.4%, 14 df, *P =* 0.0001,I^2^ = 67.8).

**Figure 3:**
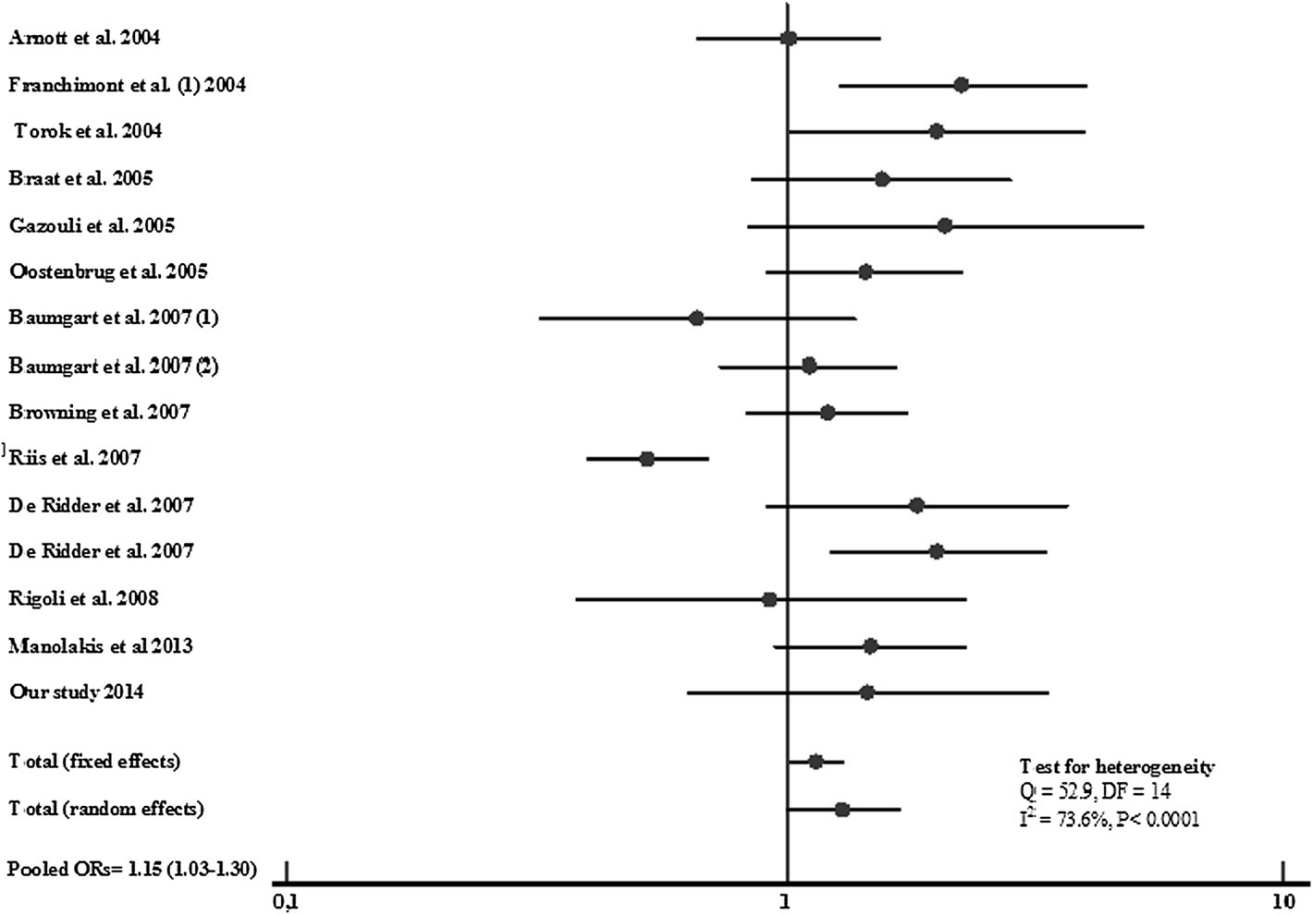
**Forest plots for the association of TLR4 A299G and risk of IBD.**

Combining Asp299Gly results for CD and UC (6115 cases and 3622 controls), an overall significant increased risk for IBD was observed, OR = 1.15, 95% CI = 1.03-1.30; P = 0.015 (Figure 3). However, a significant heterogeneity in allelic frequencies distribution is reported (Cohran’s Q = 52.9, I^2^ = 73.6%).

**Figure 4:**
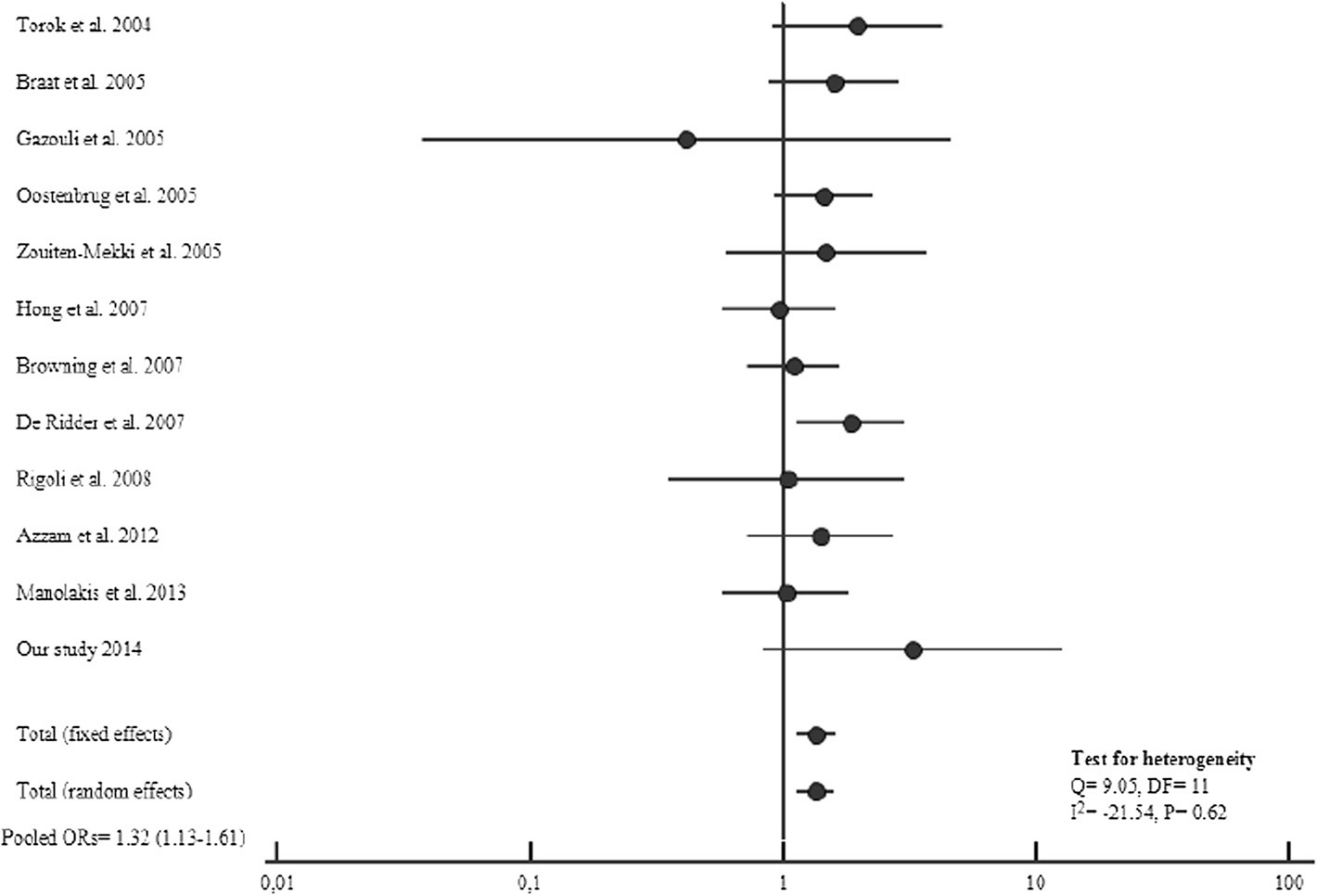
**Forest plots for the association of TLR4 T399I and risk of CD.**

**Figure 5:**
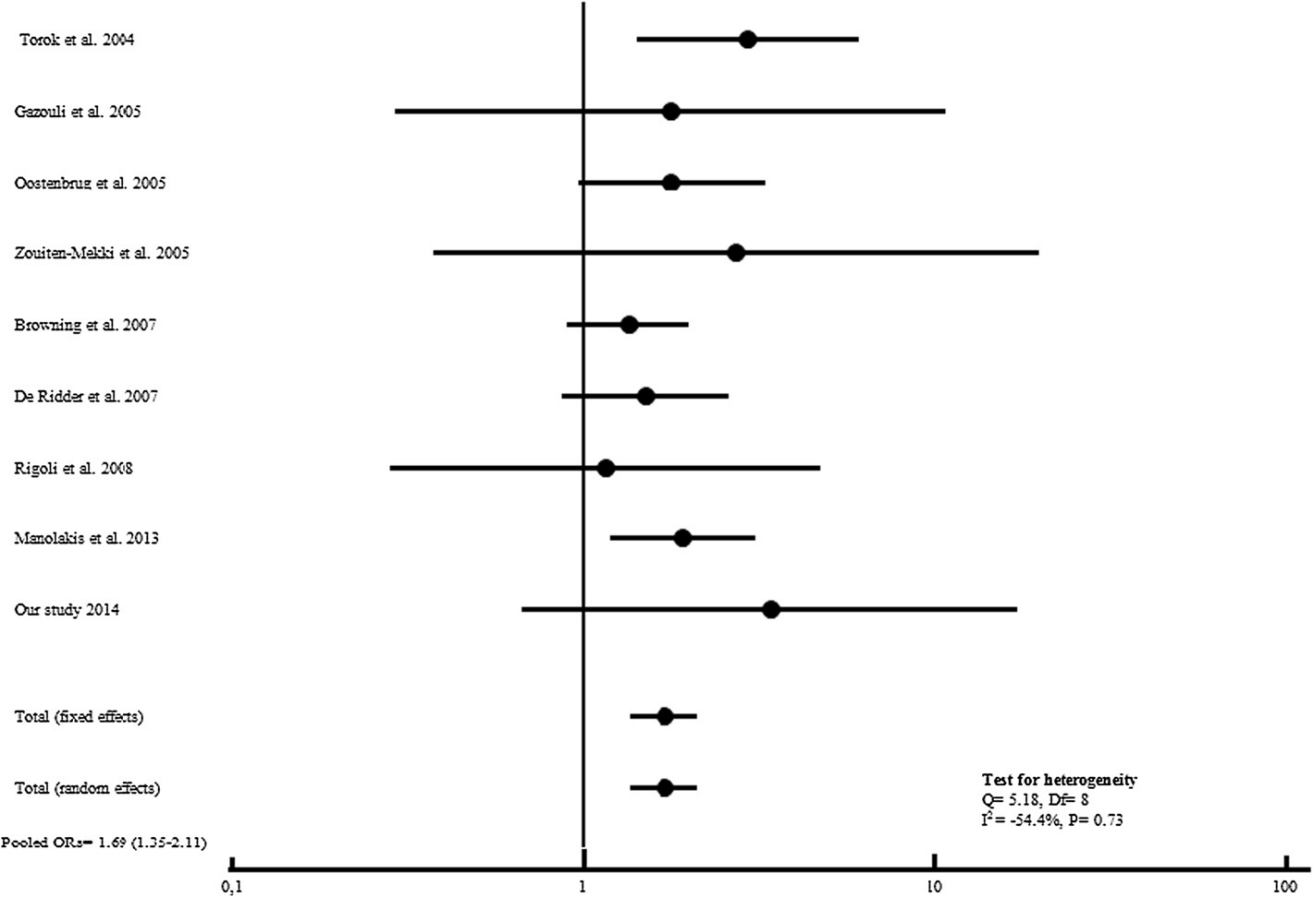
**Forest plots for the association of TLR4 T399I and risk of UC.**

**Figure 6:**
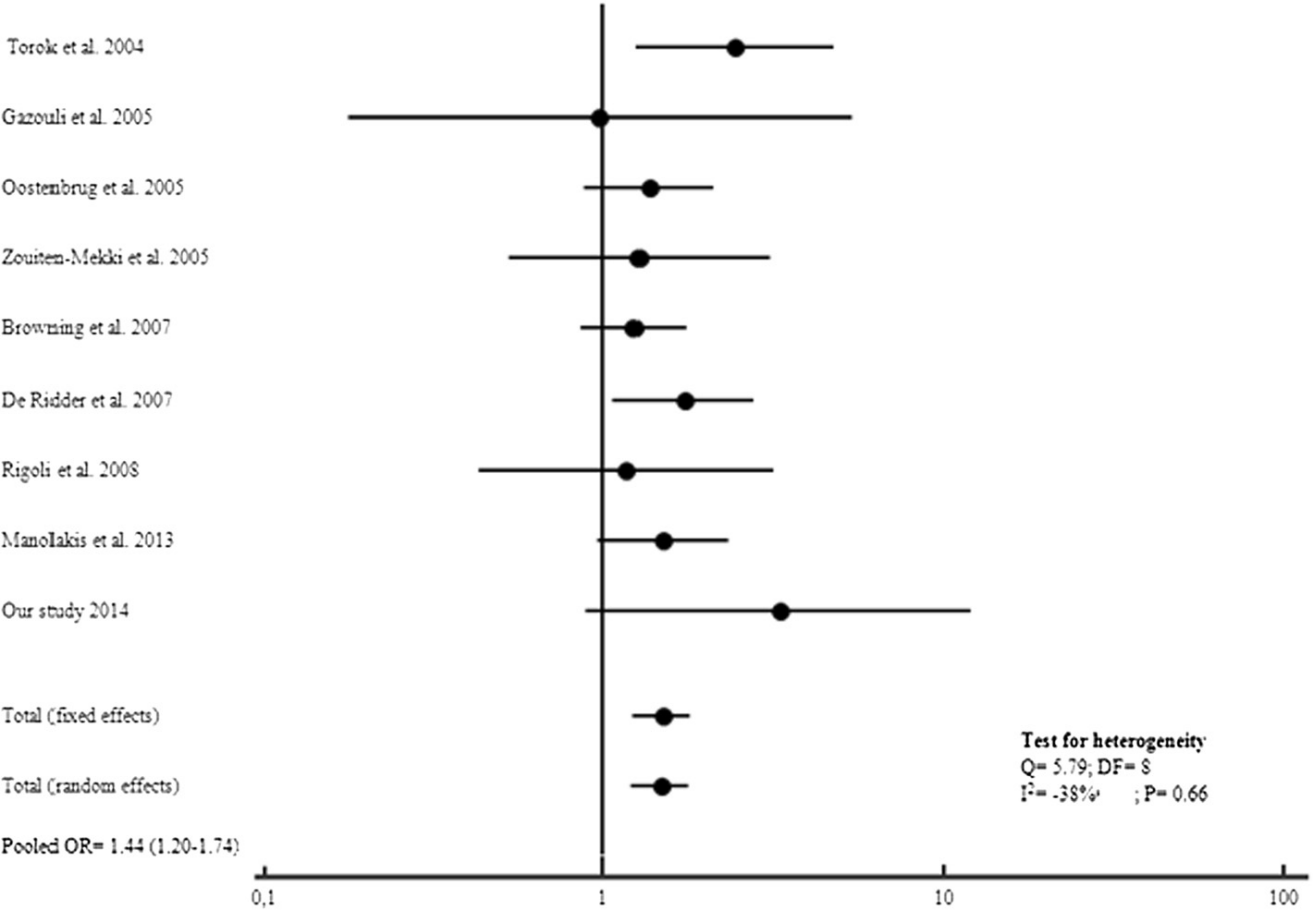
**Forest plots for the association of TLR4 T399I and risk of IBD.**

Based on the studies published so far combined to our results, we observed a significant association between the T allele of the *TLR4*Thr399Ile Polymorphism and both CD and UC risk (Figures 4 and 5). As well, *TLR4*Thr399Ile variant increased the overall IBD susceptibility when combining CD and UC results (OR = 1.46, 95%CI: 1.21-1.76; P < 0.0001) for a total of 3392 cases and 1773 controls (Figure 6).

In the present meta-analysis, we did not observed heterogeneity between studies for *TLR4*Thr399Ile Polymorphism distribution in CD (Q = 9.05, DF = 11, I^2^ = −21.54%; P = 0.62), UC (Q = 5.18, Df = 8, I^2^ = −54.4%; P = 0.73) and IBD (Cohran’sQ = 7.84, DF = 10, I^2^ = −27.5%; P = 0.64).

Genotype-phenotype correlation was investigated; demographic and clinical characteristics of CD and UC patients according to *TLR4* polymorphisms are shown in (Tables 9 and 10). A significant association was found between the need for surgery and possession of one or more Asp299gly variant alleles in UC patients (P = 0.004). The presence of *TLR4* variant alleles was not associated with smoking habits, age of diagnosis, disease location and behavior, family history and presence of extra-intestinal manifestations. Separate analyses in men and women did not reveal sex related associations. None of our UC patients had an appendectomy. The correlation study between Th399ILeu polymorphism and CD or UC didn’t allow to associate *TLR4* genotype with a particular phenotype.

**Table 9:** **Genotype-phenotype correlations in patients with Crohn’s disease**

**Parameter**	**N**	**Asp299gly (%)**			***P*** **value**	**Chi-square test**	**Thr399Ile (%)**			***P*** **value**	**Chi-square test**
		**AA**	**AG**	**GG**			**CC**	**CT**	**TT**		
Age of onset	83				0.48	1.45				0.59	1.05
<17 years	10	10 (100.0)	-				10 (100.0)	-			
17-40	63	55 (87.3.)	8 (12.7)				57 (90.5)	6 (9.5)			
>40 years	10	9 (90.0)	1 (10.0)				9 (90.0)	1 (10.0)			
Sex	83				0.35	0.87				0.17	1.92
Woman	25	24 (96.0)	1 (4.0)				25 (100.0)	-			
Man	58	50 (86.2)	8 (13.8)				51 (87.9)	7 (12.1)			
Type	83				0.54	2.14				0.36	3.23
Fistulizing	35	31 (88.6)	4 (11.4)				33 (94.3)	2 (5.7)			
Non fistulizing non stenosing	24	20 (83.3)	4 (16.7)				20 (83.3)	(16.7)			
Stenosing	17	16 (94.1)	1 (5.9)				16 (94.1)	1 (5.9)			
Fistulizing stenosing	7	7 (100.0)	-				7 (100.0)	-			
Localization	83				0.80	1.67				0.94	0.82
L1	30	26 (86.7)	4 (13.3)				27 (90.0)	3 (10.0)			
L2	20	17 (85.0)	3 (5.0)				18 (90.0)	2 (10.0)			
L3	26	24 (92.3)	2 ()				24 (92.3)	2 (7.7)			
L4	3	3 (100.0)	-				3 (100.0)	-			
L4 + L2	4	4 (100.0)	-				4 (100.0)	-			
Smoking	83				0.04	4.44				0.17	1.92
Presence	33	26 (78.8)	7 (21.2)				28 (84.8)	5 (15.2)			
Absence	50	48 (96.0)	2 (4.0)				48 (96)	2 (4.0)			
SFC	83				0.91	0.01				0.76	0.09
Presence	4	4 (100.0)	-				4 (100.0)	-			
Absence	79	70 (88.6)	9 (11.4)				72 (91.1)	7 (8.9)			
Appendectomy	83				0.84	0.04				0.58	0.30
Presence	12	11 (91.7)	1 (8.3)				10 (83.3)	2 (16.7)			
Absence	71	63 (88.7)	8 (11.3)				66 (93.0)	5 (7.0)			
EIM	83				0.97	0.001				0.97	0.001
Presence	42	38 (90.5)	4 (9.5)				39 (92.9)	3 (7.1)			
Absence	41	36 (87.8)	5 (12.2)				37 (95.1)	4 (4.9)			
Surgery	83				0.50	0.45				0.45	0.57
Presence	41	38 (92.7)	3 (7.3)				39 (95.1)	2 (4.9)			
Absence	42	36 (85.7)	6 (14.3)				37 (88.1)	5 (11.9)			

SFC: similar familial cases; EIM: extra intestinal manifestations; N: total number; AA: wild type TLR4 Asp299gly, AG: TLR4 Asp299gly heterozygous variant, GG: TLR4 Asp299gly homozygous variant; CC: wild type TLR4 Thr399Ile, CT: TLR4 Thr399Ile heterozygous variant, TT: TLR4 Thr399Ile homozygous variant.

**Table 10:** **Genotype-phenotype correlations in patients with ulcerative colitis**

**Parameter**	**N**	**Asp299gly (%)**			***P*** **value**	**Chi-square test**	**Thr399Ile (%)**			***P*** **value**	**Chi-square test**
		**AA**	**AG**	**GG**			**CC**	**CT**	**TT**		
**Age of onset**	34				0.35	0.89				0.34	2.13
**<17 years**	-	-	-				-	-			
**17-40**	20	18 (90.0)	2 (10.0)	-			19 (95.0)	1 (5.0)	-		
**>40 years**	14	10 (71.4)	4 (28.6)	-			13 (92.9)	-	1 (7.1)		
**Sex**	34				0.89	0.018				0.36	2.06
**Woman**	15	13 (86.7)	2 (13.3)	-			14 (93.3)	1 (6.7)	-		
**Man**	19	15 (78.9)	4 (21.1)	-			18 (94.7)	-	1 (5.3)		
**Extent of the disease**	34				0.31	3.55				0.16	9.34
**E1**	4	2 (50.0)	2 (50.0)	-			3 (75.0)	-	1 (25.0)		
**E2**	15	13 (86.7)	2 (13.3)	-			15 (100.0)	-	-		
**E3**	2	2 (100.0)	-	-			2 (100.0)	-	-		
**E4**	13	11 (84.6)	2 (15.4)	-			12 (92.3)	1 (7.7)	-		
**SFC**	34				0.39	0.74				0.97	0.06
**Presence**	1	1 (100.0)	-	-			1 (100.0)	-			
**Absence**	33	27 (81.8)	6 (18.2)	-			31 (93.9)	1 (3.0)	1 (3.0)		
**Smoking**	34				0.93	0.008				0.20	3.17
**Presence**	9	7 (77.8)	2 (22.2)	-			8 (88.9)	-	1 (11.1)		
**Absence**	25	21 (84.)	4 (16.0)	-			24 (96.0)	1 (4.0)	-		
**EIM**	34				0.89	0.018				0.35	2.05
**Presence**	19	15 (78.9)	4 (21.1)	-			18 (94.7)	-	1 (5.3)		
**Absence**	15	13 (86.7)	2 (13.3)	-			14 (93.3)	1 (6.7)	-		
**Surgery**	34				0.004	8.3				0.08	4.97
**Presence**	6	2 (33.3)	4 (66.7)	-			5 (83.3)	1 (16.7)	-		
**Absence**	28	26 (92.9)	2 (7.1)	-			27 (96.4)	-	1 (3.6)		

SFC: similar familial cases; EIM: extra intestinal manifestations; N: total number; AA: wild type TLR4 Asp299gly, AG: TLR4 Asp299gly heterozygous variant, GG: TLR4 Asp299gly homozygous variant; CC: wild type TLR4 Thr399Ile, CT: TLR4 Thr399Ile heterozygous variant, TT: TLR4 Thr399Ile homozygous variant.

## Discussion

Given the evidence that an altered innate immune response and chronic inflammation are implicated in IBD pathogenesis, genetic influence of pattern recognition receptors was clearly suggested as a trigger of CD and UC. Several efforts were undertaken to demonstrate associations of the human *TLR4* gene (Gene map locus 9q32-q33) with IBD and its clinical manifestation. Attention was focused on co-segregating SNPs located in exon 3 of *TLR4* causing amino acid exchanges at positions 299 (Asp299Gly) and 399 (Thr399Ile) which are located in the extracellular domain of the receptor [17,48]. Association of *TLR4* Asp299Gly with CD was first reported by Braat et al. [30] subsequent studies have had divergent results and showed strong evidence of ethnic differences. In view of the discrepant data regarding the association of the *TLR4* gene with IBD and its clinical complications, we investigated for the first time the potential influence of *TLR4 *SNPs in the susceptibility to IBD in a cohort of Moroccan patients. However, the statistical power was very low and could be considered a limitation in this study. Our study showed that the GG genotype was not found in both CD and UC patients. No significant differences were observed in allele frequencies of the *TLR4*Asp299Gly among patients and controls. In addition, although slightly increased frequencies of the mutant alleles were encountered, we were not able to identify a significant difference in allele distributions of the *TLR4*Thr399Ile in our case–control study. In line with our results, a Tunisian study that genotyped 90 patients with CD and 80 healthy individuals for the Asp299Gly and Thr399Ile polymorphisms, reported the absence of association between CD and *TLR4* gene in a north African population [40]. Although the Tunisian CD population showed a similar overall pattern of allelic frequencies, it is of some note that the genotype-phenotype correlation revealed divergent results. While the Thr399Ile variant allele was associated with early disease onset in Tunisian patients, no correlation with a particular phenotype was observed for this polymorphism in the Moroccan patients. Our study showed that the presence of Asp299Gly variant allele was associated with the need for surgery in UC patients (P = 0.004). Furthermore, the occurrence of one Asp299gly risk allele in CD patients was suggestive of a trend of association with smoking habits (P = 0.04) that was no more observed after correction for multiple testing.

Being in linkage disequilibrium, *TLR4* mutant alleles are known to be inherited in the form of Asp299Gly/Thr399Ile haplotype [48]. In a German cohort, the co-segregation between mutant alleles represented 100% in controls, whereas it was not complete in CD and UC patients: 94% and 86% respectively [33]. These observations contrast our findings where simultaneous presence of the mutated alleles was only observed in 33% of controls, 33% of UC and 60% of CD patients.

Results on the relationship of Asp299Gly SNP alone or in combination with Thr399Ile with IBD are inconsistent between studies. No difference in *TLR4* allele frequency between IBD patients and controls was observed in Hungarian [35], Saudi Arabian [47], Southern Italian [45], New Zealandian [41] and EC-IBD [43] study groups populations. Genetic heterogeneity within Europe was evidenced by Arnott et al. when reporting lack of association of *TLR4* and CD14 variants in Scottish and Irish CD patients [31]. Moreover, Baumgart DC et al. reported an association between IBD and the CD14 c.1-260C T promoter but not with the *TLR4* (p.D299G) variant in Germans and Hungarians [21]. Interestingly, the heterozygous and homozygous pattern for the mutated allele was not detected in any of the individuals from the Japanese [49], Korean [24], Chinese Han population [25] and Zhuang population from the Guangxi Zhuang Autonomous Region of China [26]. *TLR4* was linked to an increased IBD (CD or UC) risk in many other diverse investigations. Significant associations were found in patients drawn from Belgian [32], German [22,33], Greek [36,46] and Dutch [34] populations. In addition, several meta-analyses provided evidence that the Asp299Gly SNP is associated with CD and IBD in Caucasians [27,42,50]. The results of our meta-analysis indicate a significant association between TLR4 Thr399Ile and CD and UC risk in different populations. Noteworthy, a lack of heterogeneity between studies was observed regarding distribution of this polymorphism. The results support that this variant is a potential risk factor for IBD.

A correlation between Asp299Gly variant and an increased disease risk is also reported for CD and IBD but not regarding UC, which is in line with de Jager et al. findings [28]. This indicates that the risk allele is either not associated with disease susceptibility or that the small number of UC cohorts did not provide sufficient power to detect an association. A correlation between TLR4 polymorphisms and UC has been rarely discussed, more association studies are needed to validate the conclusion.

To date it is well known that the frequency of the investigated *TLR4* gene SNPs varies between populations [27,31]. Overall, there was inescapable evidence for considerable genetic heterogeneity. This observation has been explained by geographic and ethnicity-related gene effect on disease susceptibility [51]. Our results showed that the distribution of the risk alleles varies between both TLR4 polymorphisms. Therefore, we offer additional evidence for differences in the contribution of individual genetic determinants between populations. Browning et al., argued that negative studies with results that do not achieve statistical significance can still contribute evidence for association, having important implications for the first generation of whole genome association studies [42].

In view of the role of potential confounders related to the present study and to discrepant results between populations, it is likely that the contribution of different sample size, selection bias, phenotypic heterogeneity and population stratification in case control studies can’t be ruled out. These data demonstrate further the real difficulties in candidate gene analysis in complex diseases. Moreover, given that IBD is a polygenic disease it is provided that association studies will reveal various sets of susceptible genes. Therefore, further large-scale studies are required to obtain a clear insight into the impact of the pattern recognition receptors in the pathophysiological and immunogenetic aspects of IBD and to explore the contribution of other genes involved in various processes.

## Conclusions

In the present study, we have demonstrated that the common mutations in the *TLR4* gene are not associated with IBD in a sample of Moroccan patients. However, our dataset contributed to the significant association observed in TLR4 meta-analysis.

It is likely that the distribution of *TLR4* gene polymorphisms have ethnic differences. Our data suggests that other genetic and environmental factors may play a role in IBD susceptibility and behavior in this population. However, because of the relatively small sample size, additional well-powered studies are needed to confirm our findings.

## Abbreviations

IBD: Inflammatory bowel disease
CD: Crohn’s disease
UC: Ulcerative colitis
PRR: Pattern recognition receptors
